# Innovative management of hepatitis C: implementation and outcomes of the ‘SMART’ model in a general hospital

**DOI:** 10.7189/jogh.16.04241

**Published:** 2026-08-28

**Authors:** Maoping Li, Wan Hu, Zhongshang Dai, Jie Li, Jing Ma, Yi Li

**Affiliations:** The Second Xiangya Hospital, Central South University, Department of Infectious Diseases, Changsha, China

**Keywords:** Hepatitis C virus, management, general hospital, multi-disciplinary cooperation, health policy

## Abstract

**Background:**

This study aimed to evaluate the implementation and outcomes of the hepatitis C (HCV) SMART management model in a tertiary hospital, to provide insights for optimising HCV management globally.

**Methods:**

A multidisciplinary HCV working group was established at The Second Xiangya Hospital to implement an in-hospital micro-elimination strategy. Based on post-implementation analysis, the SMART Model was enhanced in July 2022 with improved HCV education, a ‘one-step’ reflexive nucleic acid testing protocol, and dedicated follow-up specialists. We conducted a retrospective analysis of patients undergoing HCV antibody and RNA testing (January 2020–December 2023), comparing three periods: the Pre-Implementation, Micro-Elimination Model, and SMART Model periods. We analysed HCV RNA testing rates and direct-acting antiviral (DAA) treatment rates using χ^2^ tests and multivariable generalised estimating equations (GEE) to account for department-level clustering.

**Results:**

A total of 355,191 patients were screened, with 4,355 (1.23%) testing anti-HCV-positive. Males and patients born before 1997 had significantly higher positivity rates. Blood-borne transmission was the predominant risk factor in the pre-1997 cohort. The Micro-Elimination Model increased the HCV RNA testing rate (53.76% *vs.* 58.33%, *P* = 0.014) but not the DAA treatment rate (65.33% *vs.* 64.21%, *P* = 0.780). In contrast, the SMART Model significantly improved both outcomes: the RNA testing rate rose to 77.10% (*P* < 0.001) and the DAA treatment rate to 71.29% (*P* = 0.044). Multivariable GEE analysis identified age <60 years, care in the Department of Infectious Diseases, and the SMART Model as positive independent predictors for both HCV RNA testing and DAA treatment initiation.

**Conclusions:**

The SMART Model significantly enhanced HCV diagnosis and treatment rates while improving clinical management. Future efforts should focus on disseminating this model to optimise healthcare resources and strategies.

Hepatitis C virus (HCV) is a blood-borne virus that primarily causes acute and chronic inflammatory lesions of the liver [1]. Approximately one-third of individuals infected with HCV spontaneously clear the virus within six months, while the remaining two-thirds progress to chronic hepatitis C (CHC). The severity of CHC can range from mild to life-threatening, and it may lead to serious complications such as liver cirrhosis and hepatocellular carcinoma [2,3].

Data from the World Health Organization (WHO) in 2020 indicated that the global prevalence of hepatitis C is approximately 0.7%, while in China, this figure stands at around 9.6 million [4]. Currently, the diagnosis of hepatitis C in China primarily relies on serological tests and viral load assays [5]. However, the lack of effective screening strategies results in many infected individuals remaining undiagnosed in the early stages, allowing the disease to progress to advanced stages [6,7]. In response, the World Health Organization (WHO) has set a goal to eliminate hepatitis C as a public health problem by 2030, with targets to diagnose 90% of people with HCV infection and treat 80% of those diagnosed [8]. To date, however, screening and treating hepatitis C-infected individuals remain a pressing global challenge [9]. Limited resources, coupled with insufficient awareness of hepatitis C among healthcare providers and the general public, have posed significant obstacles to comprehensive screening and treatment efforts. Strengthening surveillance among specific high-risk populations and implementing a ‘micro-elimination’ strategy may serve as an effective approach to control hepatitis C [10,11]. Existing studies have demonstrated that systematic in-hospital screening and treatment can significantly reduce HCV transmission, alleviate healthcare burdens, and improve health outcomes [12,13]. However, the ‘micro-elimination’ model has certain limitations: it targets only local or specific populations, and long-term follow-up and monitoring systems remain inadequate, among other issues. Consequently, there remains a lack of effective in-hospital hepatitis C management models to guide patients and healthcare providers through the cascaded care process of ‘screening, diagnosis, and treatment’.

Given the existing gaps in hepatitis C management in China, we have further refined and innovatively developed a distinctive in-hospital hepatitis C SMART management model for general hospitals, building upon the ‘micro-elimination” framework. The specific measures are as follows: S (Screen): clinicians recommend and conduct screening for hepatitis C antibodies among inpatients and outpatients with high-risk factors. M (Manage): collaborative data management is implemented by the Department of Information Technology, Department of Laboratory Medicine, and other relevant departments. A key component is the introduction of a ‘one-step hepatitis C nucleic acid testing assay’, which significantly streamlines the process. A (Act): an automated alert system is established to automatically send text notifications to attending physicians and patients, informing them of test results and prompting subsequent actions (*e.g.* RNA testing or referral) to ensure procedural adherence. R (Refer): a streamlined referral process is established – for outpatients, the treating physician completes a referral form to the Department of Infectious Diseases, and for inpatients, the attending physician in charge submits a consultation request form for evaluation by physicians from the Department of Infectious Diseases. T (Treat): physicians in the Department of Infectious Diseases are responsible for pretreatment evaluation and formulating treatment plans. Dedicated follow-up specialists are assigned to oversee antiviral therapy monitoring, post-treatment follow-up, and active recall of untreated patients. The SMART Model is continuously refined in clinical practice, aiming to enhance the efficiency of screening, diagnosis, and treatment, while optimising the utilisation of healthcare resources.

Thus, this study aims to explore the implementation and outcomes of the hepatitis C SMART management model in a tertiary general hospital, providing insights and references for medical institutions nationwide and globally, as well as offering a novel strategy for optimising hepatitis C management and conserving healthcare resources.

## METHODS

### Formulation of the hepatitis C elimination strategy

In March 2021, The Second Xiangya Hospital of Central South University established a multidisciplinary hepatitis C task force guided by the Medical Affairs Department and led by the Department of Infectious Diseases, in collaboration with the Department of Laboratory Medicine, Blood Transfusion Division, Blood Purification Center, and other relevant clinical departments. This task force developed and implemented a standardised in-hospital management system for hepatitis C, termed the micro-elimination strategy.

**Figure 1:**
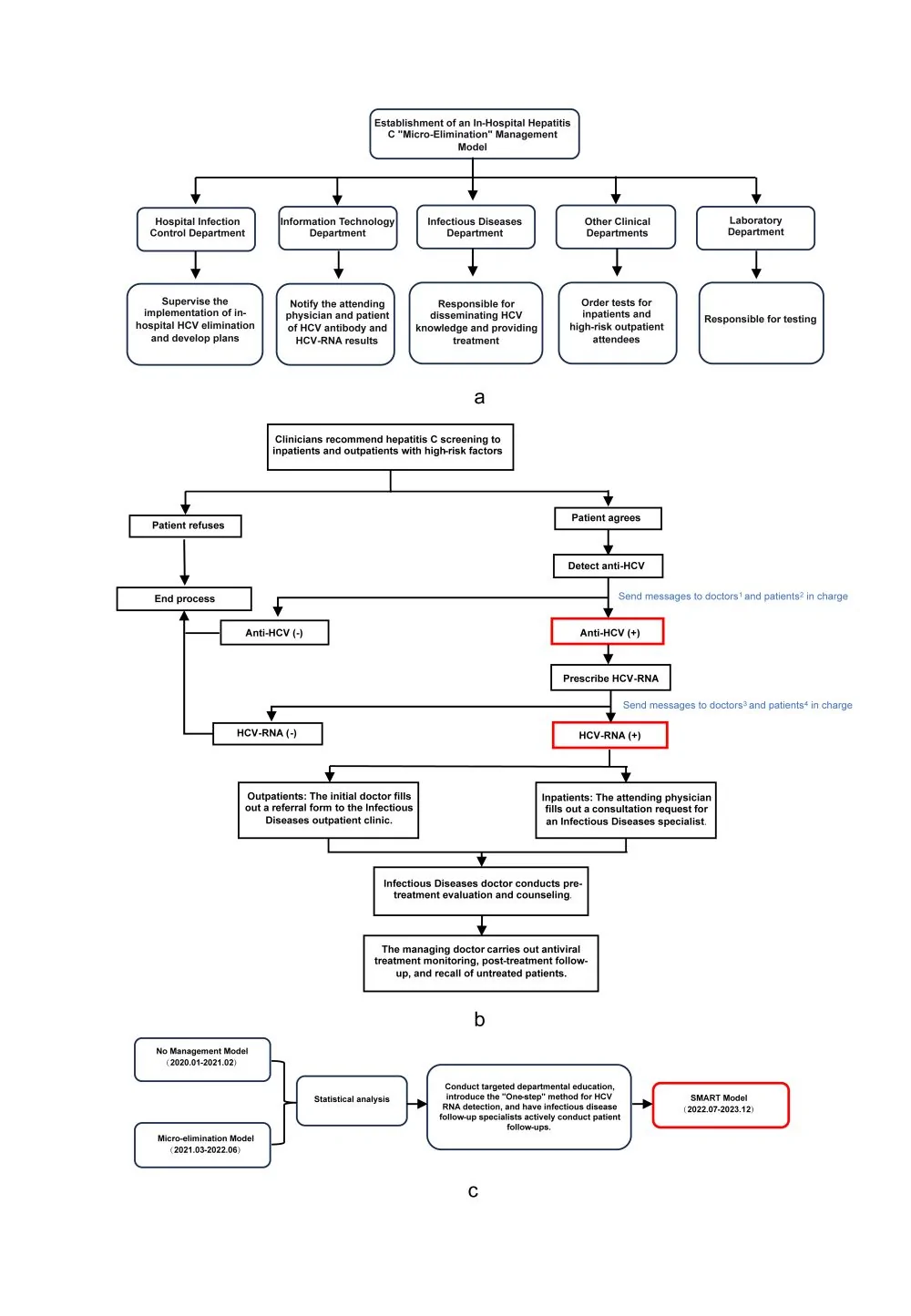
Development and implementation of the in-hospital SMART management model for hepatitis C. Panel A. Micro-Elimination Management model. Panel B. Optimised in-hospital SMART Model for hepatitis C. Panel C. Construction process of the SMART Model. *Dr (XXX), patient (XX) (Hospitalisation ID/Patient Number: (XXXXX)) has tested positive for HCV antibody. Please order HCV RNA testing to confirm the diagnosis. †Mr/Ms (XX), your HCV antibody test result is positive. We recommend you proceed with HCV RNA testing to confirm the diagnosis. ‡Dr (XX), patient (X) (Hospitalisation ID/Patient Number: (XXXXX)) has tested positive for HCV RNA. Please complete the infectious disease report card and request an infectious diseases consult or refer the patient to the infectious diseases department. HCV – hepatitis C virus, RNA – ribonucleic acid.

Within this framework, the Hospital Infection Control Department oversaw the overall implementation of HCV elimination initiatives and developed related plans. The Information Technology Department utilised the hospital's information system to send automated notifications of HCV antibody and HCV RNA test results to both the attending physicians and patients upon test completion. The Department of Infectious Diseases was responsible for disseminating knowledge about HCV and providing standardised treatment for diagnosed patients. Other clinical departments were tasked with ordering HCV-related tests for inpatients and high-risk outpatients. The Clinical Laboratory performed all laboratory testing for HCV antibodies and HCV RNA. With clearly defined and coordinated roles, these departments collectively formed the management system for the hospital's hepatitis C micro-elimination. The specific responsibilities of this collaborative group are detailed in **Figure 1**, Panel A.

Based on an analysis comparing pre- and post-implementation of the micro-elimination strategy, a revised plan – the SMART Model – was proposed in July 2022. This model enhanced the original strategy with the following additions:

• Intensified education on hepatitis C knowledge targeted at departments with lower implementation rates.

• After implementation of the SMART management model, a reflex HCV RNA testing protocol was introduced in the hospital laboratory system. When a patient tested positive for anti-HCV antibody, the laboratory automatically performed HCV RNA testing on the same serum sample without requiring an additional blood draw. Serum samples collected for anti-HCV testing were retained and processed for reflex RNA testing if the antibody result was positive. Detection of HCV RNA was performed using a commercial real-time PCR assay approved for clinical use. This reflex testing protocol was implemented hospital-wide and applied consistently across inpatient and outpatient departments to reduce diagnostic delays and minimise loss of patients between screening and confirmatory testing.

• The appointment of dedicated HCV follow-up specialists within the Department of Infectious Diseases. These specialists are responsible for monitoring antiviral treatment and conducting follow-ups for diagnosed patients, both from their own department and those referred from other clinics, and actively initiating consultations and recalls for untreated patients.

The resulting management flowchart for HCV antibody-positive patients is schematically represented in **Figure 1**, Panel B. This completed the development process of the SMART Model (**Figure 1**, Panel C).

### Data and analysis

We conducted a retrospective analysis of all patients who underwent HCV antibody and HCV RNA testing at the Second Xiangya Hospital of Central South University between 1 January 2020 and 31 December 2023, as presented in Figure S1 in the **Online Supplementary Document**. Databases were established for anti-HCV-positive and HCV RNA-positive patients, which included basic demographic and clinical information such as patient identifiers, contact details, and corresponding clinical departments and diagnoses. Patient identifiers were temporarily retained solely by the designated infectious disease follow-up specialists for the purpose of structured telephone follow-ups. Upon completion of outcome ascertainment, all data sets were immediately de-identified and pseudo-anonymised prior to statistical analysis. Only de-identified data were accessible to the data analysis team. Duplicate records were identified using unique patient identifiers. For patients with multiple encounters during the study period, records were consolidated at the patient level. The first anti-HCV-positive result was defined as the index event, and subsequent information on HCV RNA testing and DAA treatment initiation was obtained through review of medical records and telephone follow-up. This approach allowed us to capture whether patients eventually received RNA testing or treatment during the study period. Patients with positive anti-HCV antibody results during the study period were included in the analysis. HCV RNA testing rate was defined as the proportion of anti-HCV-positive patients who subsequently underwent HCV RNA testing. The DAA treatment rate was defined as the proportion of HCV RNA-positive patients who initiated DAA therapy.

During the data collection process, telephone follow-up was conducted to ascertain the final treatment status of RNA-positive patients. Among the 998 RNA-positive patients, 336 (33.6%) experienced follow-up failure (*e.g.* phone not connected, wrong number, or refused to answer). Given the inability to confirm their clinical outcomes, we adopted a conservative complete-case analysis approach where these patients with missing follow-up data were classified as ‘untreated’. This worst-case scenario assumption prevents overestimating the intervention effect of the SMART model.

We compared the HCV RNA testing rate among anti-HCV-positive patients and the treatment rate among HCV RNA-positive patients across three periods: the Pre-Implementation period (January 2020–February 2021), the Micro-Elimination Model period (March 2021–June 2022), and the SMART Model period (July 2022–December 2023). The index date was defined as the date of the first positive anti-HCV encounter. Any RNA test following the index antibody positivity prior to the end of the study period was captured. The HCV RNA testing rate was calculated as the percentage of anti-HCV-positive patients who underwent HCV RNA testing. The treatment rate was defined as the percentage of HCV RNA-positive patients who received DAA therapy. The treatment denominator includes all RNA-positive results, as our hospital protocol initiates pan-genotypic DAA treatment for all confirmed viremic patients to maximise linkage to care.

The primary outcomes of this study were HCV RNA testing among anti-HCV-positive patients and initiation of DAA therapy among HCV RNA-positive patients. Both outcomes were binary variables and were expressed as proportions. Therefore, χ^2^ tests were used to compare differences in RNA testing rates and treatment rates across the three management periods (No Management, Micro-elimination, and SMART model).

To further identify factors independently associated with HCV RNA testing and DAA treatment initiation while accounting for department-level clustering, multivariable generalised estimating equation (GEE) models were performed. Covariates included age group, sex, patient type (inpatient or outpatient), clinical department (with ‘Department of Infectious Diseases’ strictly referring to the patient's initial physical location at the time of baseline screening), and management period. Odds ratios (ORs) and 95% confidence intervals (CIs) were calculated to estimate the strength of associations between these variables and the study outcomes.

A two-sided *P*-value <0.05 was considered statistically significant. All analyses were conducted using IBM SPSS Statistics for Windows, version 26.0 (IBM Corp., Armonk, NY, USA), using the Generalized Estimating Equations procedure.

## RESULTS

### Demographic characteristics of anti-HCV-positive patients

From January 2020 to December 2023, a total of 355,191 patients were screened for HCV at our hospital. Among them, 4,355 patients were identified as anti-HCV positive, including 2,383 males (49.50%). The overall anti-HCV positivity rate was 1.23%. The positivity rate was significantly higher in male patients than in females (1.36% *vs.* 1.10%; χ^2^ = 48.99, *P* < 0.001). The age of anti-HCV-positive patients ranged from 1 day to 97 years, with a median age of 54 years (interquartile range (IQR): 46–63 years).

**Figure 2:**
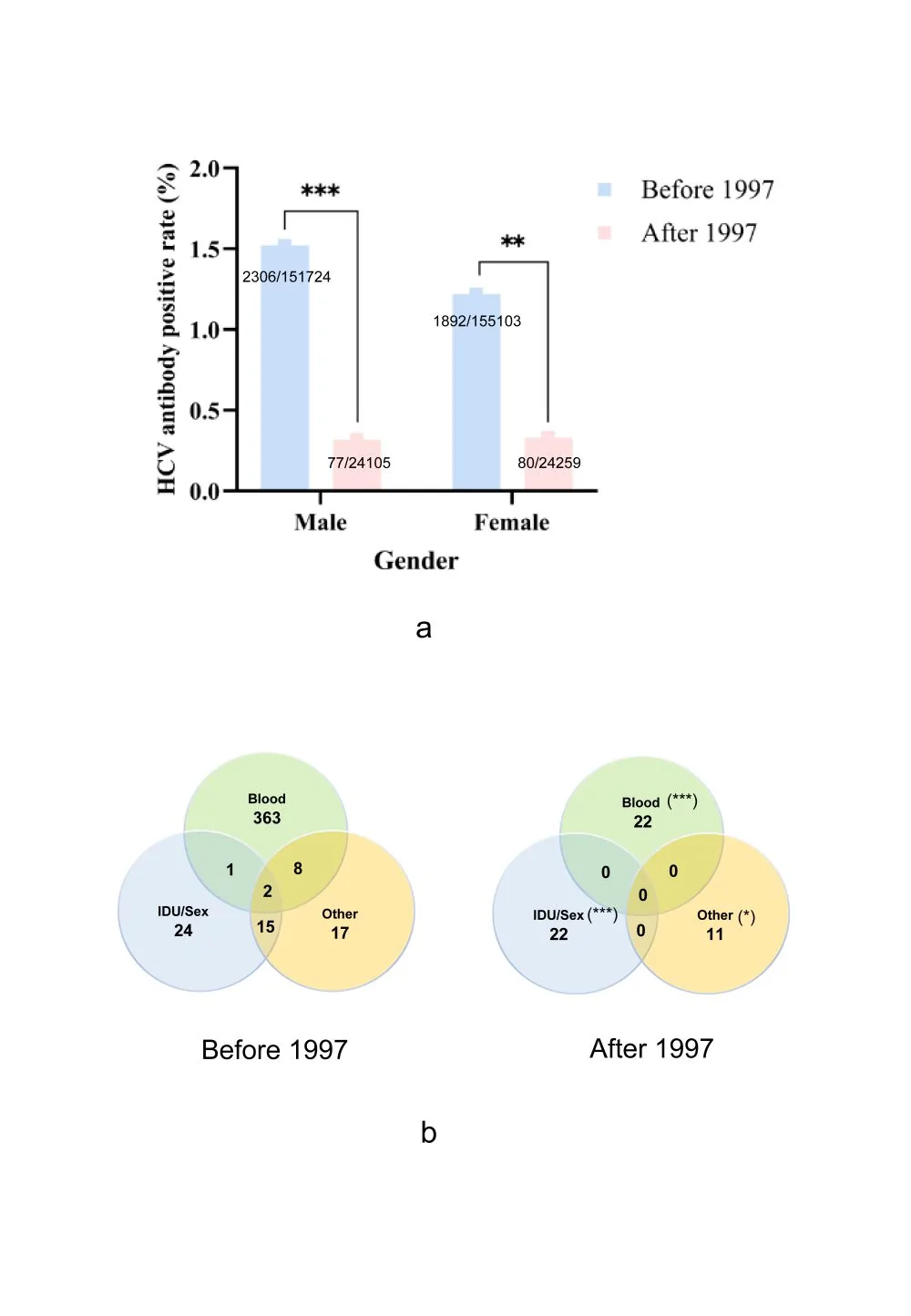
Panel A. HCV antibody positivity rate by birth year. Panel B. Distribution of high-risk factors among hospitalised patients with positive HCV antibody. HCV – hepatitis C virus.

When stratified by year of birth, patients born before 1997 exhibited a significantly higher anti-HCV positivity rate compared to those born in or after 1997 (1.37% *vs.* 0.33%, *P* < 0.01). The decline in seropositivity was more pronounced among male patients than females, as illustrated in **Figure 2**, Panel A. Among the anti-HCV positive individuals, 2,865 were inpatients. An analysis of HCV-related high-risk factors revealed that, compared to seropositive individuals born before 1997, those born in or after 1997 were significantly more likely to have risk factors such as drug use or sexual transmission (9.77% *vs.* 40.00%, *P* < 0.001). In contrast, blood-borne transmission remained the predominant risk factor in the pre-1997 birth cohort (86.98% *vs.* 40.00%, *P* < 0.001), as shown in **Figure 2**, Panel B.

Of the 4355 patients who screened positive for anti-HCV, 2766 underwent HCV RNA testing, yielding an overall testing rate of 63.51%. Testing was more common among inpatients than outpatients. Patients managed in the Department of Infectious Diseases were more likely to receive confirmatory HCV RNA testing, with a testing rate of 87.91%, compared to 56.39% in other clinical departments (Table S1 in the **Online Supplementary Document**). Among the 2766 patients tested for HCV RNA, 998 tested positive. Of these, 674 received DAA treatment, resulting in an overall treatment rate of 67.54%. χ^2^ analysis indicated that patients who were managed in the Department of Infectious Diseases, those treated in an outpatient setting, and those under 60 years of age were significantly more likely to initiate DAA therapy.

We compared the baseline characteristics of anti-HCV-positive patients across the three management periods to evaluate potential case-mix changes. Sex distribution differed slightly across periods (*P* < 0.001), and department composition also showed a modest difference (*P* = 0.012). However, inpatient status, age group, and the proportion of patients managed in the Infectious Diseases Department were comparable across periods (*P* > 0.05). Importantly, standardised mean differences for all variables were small (SMD<0.1), suggesting that the overall case-mix of anti-HCV–positive patients remained broadly similar across the three periods.

### Evaluation of hepatitis C management strategies

Following the implementation of the Micro-Elimination Model, the HCV RNA testing rate demonstrated a significant increase (53.76% *vs.* 58.33%, *P* = 0.014). However, no statistically significant difference was observed in the DAA treatment rate during this period (65.33% *vs.* 64.21%, *P* = 0.780). In response to these findings, we developed the hospital's SMART model, which introduced key enhancements: intensified in-hospital education on hepatitis C, the adoption of a ‘one-step’ reflexive nucleic acid testing protocol (whereby HCV RNA testing is automatically performed on the same sample following a positive antibody result, eliminating the need for a second blood draw), and the assignment of dedicated case managers for HCV patient follow-up. The implementation of the SMART Model led to further significant improvements. The HCV RNA testing rate increased from 58.33% to 77.10% (*P* < 0.001), and the DAA treatment rate rose from 64.21% to 71.29% (*P* = 0.044). These results indicate that the refined SMART Model has significantly improved the effectiveness of our in-hospital hepatitis C management programme (**Table 1**).

**Table 1:** Changes in hospital-wide HCV RNA testing and DAA treatment rates before and after implementation and optimisation of the hepatitis C management model

	No management, % (n/N)	Micro-elimination, % (n/N)	SMART, % (n/N)	*P*-value	No management
RNA testing rate	53.73 (728/1,355)	59.29 (941/1,587)	77.64 (1,097/1,413)	0.002	<0.001
DAA treatment rate	65.33 (179/274)	64.44 (212/329)	71.65 (283/395)	0.820	0.038

DAA – direct-acting antiviral. HCV – hepatitis C virus, RNA – ribonucleic acid

The monthly HCV RNA testing rates and the proportion of patients receiving DAA therapy after testing are presented in Figure S2 in the **Online Supplementary Document**, which serves as a sensitivity analysis to assess the robustness of the SMART Model implementation. Both the number of patients tested (blue bars) and the post-test treatment rates (red line) steadily increased over the study period. Notable improvements were observed following the introduction of the micro-elimination strategy in March 2021 and the SMART management model in July 2022, as indicated by the vertical dashed lines. These monthly trends confirm that the observed increases in RNA testing and treatment rates are consistent when examined at a finer temporal resolution, supporting the robustness of the intervention effect beyond the three-period average comparisons.

Next, we analysed the changes in HCV RNA testing rates and treatment rates across different departments under various management models, as shown in Table S2 in the **Online Supplementary Document**. The data revealed that in the Infectious Diseases Department, the RNA testing rates remained high (approximately 86%–89%) across all three models, with no significant differences. In contrast, in other departments, the SMART Model significantly increased the RNA testing rate from 44.47% in the No Management group to 74.47%. Regarding treatment, the SMART Model also performed best, achieving the highest DAAs treatment rates in both the Infectious Diseases Department and other departments (92.62% and 62.27%, respectively), with statistically significant improvements. This indicates that the SMART Model effectively enhances the testing rate in non-Infectious Diseases Departments and comprehensively promotes patient treatment rates.

**Figure 3:**
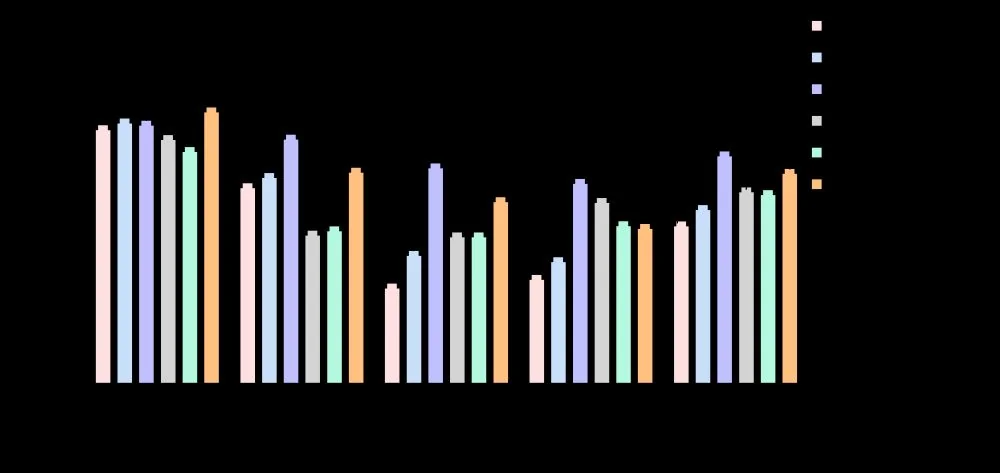
Changes in HCV RNA testing rates and DAA treatment rates across departments under different management models. DAA – direct-acting antiviral, HCV – hepatitis C virus, RNA – ribonucleic acid.

Furthermore, we performed a subanalysis by categorising the departments into Infectious Diseases, Internal Medicine, Surgery, and Others. Departments implementing the SMART Model generally demonstrated the highest HCV RNA testing and DAA treatment rates across these departmental categories. Internal Medicine departments also consistently exhibited higher HCV RNA testing and DAA treatment rates than Surgical departments, regardless of the management model applied (**Figure 3**).

### Factors associated with in-hospital hepatitis C management

Patient diagnoses and treatment status were evaluated through medical record review and telephone follow-up (**Table 2**, **Table 3**). Multivariable GEE analysis identified that age <60 years (OR = 1.17; 95% CI = 1.03–1.32), receiving care in the Department of Infectious Diseases (OR = 8.89; 95% CI = 6.16–12.85), and management under the SMART Model (OR = 3.13; 95% CI = 2.20–4.47) were independent factors associated with undergoing HCV RNA testing.

**Table 2:** Factors associated with undergoing HCV RNA testing among patients (GEE model)

Variable	Univariate GEE OR (95% CI)	*P*-value	Multivariable GEE OR (95% CI)	*P*-value
Male	1.09 (0.87–1.37)	0.431	1.02 (0.83–1.26)	0.826
Age <60 years	1.18 (0.90–1.55)	0.221	1.17 (1.03–1.32)	0.014
Inpatient	0.90 (0.34–2.37)	0.837	2.08 (1.27–3.41)	0.004
Infectious Diseases Dept	5.62 (4.32–7.32)	<0.001	8.89 (6.16–12.85)	<0.001
In-hospital HCV management model				
No management	1 (Reference)		1 (Reference)	
Micro-elimination	1.20 (1.05–1.38)	0.007	1.23 (1.04–1.44)	0.012
SMART	2.90 (1.99–4.23)	<0.001	3.13 (2.20–4.47)	<0.001

CI – confidence interval, GEE – generalised estimating equations, HCV RNA – hepatitis C virus ribonucleic acid, OR – odds ratio

**Table 3:** Factors associated with initiation of DAAs treatment in hepatitis C patients (GEE model)

Variable	Univariate GEE OR (95% CI)	*P*-value	Multivariable GEE OR (95% CI)	*P*-value
Male	0.82 (0.66–1.01)	0.064	0.82 (0.64–1.04)	0.099
Age <60 years	1.74 (1.39–2.18)	<0.001	1.58 (1.20–2.08)	0.001
Inpatient	0.47 (0.32–0.70)	<0.001	0.93 (0.38–2.27)	0.880
Infectious Diseases Dept	4.17 (3.44–5.06)	<0.001	4.14 (2.45–7.01)	<0.001
In-hospital HCV management model				
No Management	1 (Reference)		1 (Reference)	
Micro-elimination	0.95 (0.74–1.23)	0.709	0.86 (0.62–1.19)	0.361
SMART	1.32 (0.93–1.87)	0.124	1.62 (1.14–2.31)	0.007

CI – confidence interval, DAA – direct acting antiviral, GEE – generalised estimating equations, HCV – hepatitis C, OR – odds ratio

For DAA treatment initiation, age <60 years (OR = 1.58; 95% CI = 1.20–2.08), care in the Department of Infectious Diseases (OR = 4.14; 95% CI = 2.45–7.01), and management under the SMART Model (OR = 1.62; 95% CI = 1.14–2.31) were identified as significant independent correlates. Notably, younger patients (aged <60 years) were significantly more likely to receive antiviral treatment. While inpatient status was negatively associated with treatment initiation in the univariate analysis, it lost statistical significance after adjustment for other variables in the multivariable GEE model.

## DISCUSSION

To address global challenges in HCV management and WHO’s 2030 elimination goals, we developed the in-hospital SMART Model (Screen, Manage, Act, Refer, Treat) based on ‘micro-elimination’. A retrospective analysis (January 2020–December 2023, 355,191 screened patients) showed SMART significantly boosted HCV RNA testing rate (58.33% to 77.10%, *P* < 0.001) and DAA treatment rate (64.21% to 71.29%, *P* = 0.044) *vs.* prior strategies. Key enablers included ‘one-step’ nucleic acid testing and dedicated follow-up. The model enhances in-hospital HCV care, offering a scalable strategy for global institutions.

Retrospective analysis data showed that among 4355 HCV antibody-positive cases, the positivity rate was higher in male patients than in female patients, which is consistent with the findings of multiple previous studies [14–17]. This may be attributed to the higher prevalence of high-risk factors such as high-risk sexual behaviours and intravenous drug use among males. In contrast, the HCV antibody positivity rate was lower in the population aged <40 years, potentially linked to the implementation of HCV antibody screening in China starting in 1993 and the standardised management of blood products initiated in 1997 [18,19]. These measures effectively reduced iatrogenic infections transmitted through blood transfusion and haemodialysis. In fact, many hepatitis C patients have a history of blood transfusion or surgery before 1997; among HCV antibody-positive patients in our hospital, 50.053% had a definite history of surgery or blood transfusion prior to admission, reflecting the historical weaknesses in blood safety management in China. However, advancements in testing technologies, continuous improvement of laws and regulations, widespread use of disposable syringes, and enhanced management have collectively controlled blood-borne hepatitis C infections in China.

Since we first implemented the hepatitis C Micro-Elimination Model in our hospital, we observed an improvement in the diagnosis rate of hepatitis C, but the treatment rate remained suboptimal. To address this, we further introduced a series of optimisation measures, including refining medical management protocols, enhancing hepatitis C awareness campaigns, adopting the ‘one-step’ nucleic acid test, and assigning dedicated personnel to patient management – collectively forming the SMART management model. Following its implementation, the HCV RNA testing rate increased from 58% to 77%, and the DAA treatment rate rose from 64% to 71%, demonstrating the significant efficacy of the SMART model. Notably, the ‘one-step’ nucleic acid test emerged as a key measure in improving testing rates. It automatically performs nucleic acid verification after initial positive antibody screening, eliminating the need for repeated sampling, strengthening the linkage between screening and confirmation, and reducing patient loss. This method integrates sample lysis, nucleic acid extraction, and amplification steps into a fully enclosed, automated process. With a single sample addition, the test can be completed, shortening the turnaround time from 4–6 hours to 1.5–2 hours, which may improve the efficiency of confirmatory testing and reduce patient loss between screening and diagnosis, making it particularly suitable for samples with low viral loads [20]. Importantly, baseline characteristics of anti-HCV–positive patients were largely comparable across the three management periods, suggesting that the observed improvements in RNA testing and treatment rates were unlikely to be driven by major case-mix differences.

In the standardised treatment of hepatitis C, patients with positive antibodies are required to make multiple hospital visits to complete RNA quantification, genotyping, and fibrosis assessment before initiating treatment. These procedures increase time costs and reduce treatment adherence, which may explain why the treatment rate of inpatients in non-infectious disease departments of our hospital is lower than that of outpatients. Liver cirrhosis comorbidity is identified as one of the risk factors for DAA treatment failure [21]. Therefore, a simplified protocol can be implemented for patients with mild conditions: upon confirmation of RNA positivity, pan-genotypic antiviral treatment is initiated immediately, with concurrent genotyping testing; subsequent treatment adjustments are guided remotely based on test results. This strategy is expected to reduce the number of hospital visits for patients and improve their willingness to undergo treatment. Furthermore, the lack of a unified infectious disease reporting system and insufficient information sharing across medical institutions in China mean that a patient’s prior infectious disease history cannot be promptly retrieved when they seek care at different hospitals [22]. This often leads to missed diagnoses, repeated testing, and treatment dropout, increasing the burden on patients and reducing treatment adherence. Additionally, existing systems struggle to update and track treatment progress in real time. Compounded by the fact that patients can obtain medications from external pharmacies under the ‘dual-channel medical insurance’ policy, the difficulty of full-course supervision is further heightened [1]. To address these challenges, we propose collaborating with centres for disease control and prevention (CDCs): dedicated personnel would be assigned to recall patients with positive RNA results, urge them to seek medical care nearby, and upload treatment information to a visualised system accessible province-wide or nationwide. This initiative aims to establish a hepatitis C elimination network, strengthen follow-up management, and ensure that all eligible patients receive treatment.

After the implementation of the hepatitis C SMART management model in our hospital, both the HCV RNA testing rate and treatment rate among patients have significantly improved. However, there remains a substantial gap from the viral hepatitis control targets set by the WHO. During the implementation of the SMART management model, several factors that may affect management efficiency persist. Studies have indicated that physicians’ professional experience and knowledge of hepatitis C can significantly influence patients’ healthcare-seeking behaviours and willingness to receive treatment [23–25]. Additionally, the general public’s lack of awareness that hepatitis C is curable may lead to fear of the disease among the general population, thereby fostering reluctance to seek medical care. A foreign study reported that only 47.3% of participants were aware that the hepatitis C virus is curable; this lack of awareness may also hinder the implementation of management models [26]. Furthermore, other studies have shown that approximately one-third of hepatitis C patients suffer from depression, with an incidence 1.5 to 4 times higher than that in patients with chronic hepatitis B or the general population. This high depression rate is associated with lower average monthly household income, comorbidity with other diseases, and the presence of liver discomfort [27]. Therefore, enhancing public awareness of the curability of hepatitis C is crucial for early intervention, reducing disease transmission, and alleviating the societal healthcare burden.

This study has several limitations. First, as a single-centre retrospective study, it has a relatively homogeneous sample source, which may restrict the external validity of the model. Second, regarding the evaluation of implementation effects, we were unable to employ an interrupted time-series (ITS) framework with finer time granularity (*e.g.* monthly or quarterly) due to data sparsity and statistically unstable estimates at smaller time intervals across different departments. Third, we could not perform time-to-event analyses (*e.g.* Kaplan-Meier or Cox models) or calculate precise intermediate process metrics (such as the exact time from antibody positivity to RNA testing or DAA initiation). This was primarily because precise timestamps were not consistently recorded; many patients obtained DAA medications from external pharmacies under the regional ‘dual-channel medical insurance’ policy, and treatment confirmation often relied on telephone follow-ups yielding unspecific dates. Fourth, detailed implementation fidelity metrics for the ‘one-step’ reflex testing protocol (*e.g.* exact proportions of sample inadequacy or haemolysis rejection rates) were not systematically retained in our retrospective laboratory database. Finally, relying on telephone follow-ups may introduce certain information biases; however, we applied a conservative complete-case analysis assumption (classifying uncontactable patients as untreated) to minimise the overestimation of the SMART model's treatment outcomes. Future prospective, multicentre studies with rigorous longitudinal tracking are warranted to validate these findings.

## CONCLUSIONS

The innovative implementation of our SMART Model has significantly improved the diagnosis and treatment rates of hepatitis C, while enhancing the level of clinical management for hepatitis C within the hospital. In the future, this model will be promoted to more general hospitals to optimise healthcare resources and strategies.

## Additional material


Online Supplementary Document

## Data Availability

**Data availability:** The de-identified minimum data set underpinning the findings of this study, along with the statistical analysis code used, are available from the corresponding author upon reasonable request. The raw data are not publicly available due to patient confidentiality concerns.

## References

[1] Shepard CW Finelli L Alter MJ Global epidemiology of hepatitis C virus infection. Lancet Infect Dis. 2005;5:558–67. 10.1016/S1473-3099(05)70216-4 16122679

[2] Westbrook RH Dusheiko G Natural history of hepatitis C. J Hepatol. 2014;61(1 Suppl):S58–68. 10.1016/j.jhep.2014.07.012 25443346

[3] Ishikawa T Strategy for improving survival and reducing recurrence of HCV-related hepatocellular carcinoma. World J Gastroenterol. 2013;19:6127–30. 10.3748/wjg.v19.i37.6127 24115808 PMC3787341

[4] Polaris Observatory HCV Collaborators Global change in hepatitis C virus prevalence and cascade of care between 2015 and 2020: a modelling study. Lancet Gastroenterol Hepatol. 2022;7:396–415. 10.1016/S2468-1253(21)00472-6 35180382

[5] Yue T Zhang Q Cai T Xu M Zhu H Pourkarim MR Trends in the disease burden of HBV and HCV infection in China from 1990-2019. Int J Infect Dis. 2022;122:476–85. 10.1016/j.ijid.2022.06.017 35724827

[6] Tanaka M Katayama F Kato H Tanaka H Wang J Qiao YL Hepatitis B and C virus infection and hepatocellular carcinoma in China: a review of epidemiology and control measures. J Epidemiol. 2011;21:401–16. 10.2188/jea.JE20100190 22041528 PMC3899457

[7] Ramos H Linares P Badia E Martín I Gómez J Almohalla C Interferon-free treatments in patients with hepatitis C genotype 1-4 infections in a real-world setting. World J Gastrointest Pharmacol Ther. 2017;8:137–46. 10.4292/wjgpt.v8.i2.137 28533924 PMC5421113

[8] World Health Organization. Global health sector strategy on viral hepatitis 2016–2021: towards ending viral hepatitis. Geneva: World Health Organization; 2016.

[9] Usuda D Kaneoka Y Ono R Kato M Sugawara Y Shimizu R Current perspectives of viral hepatitis. World J Gastroenterol. 2024;30:2402–17. 10.3748/wjg.v30.i18.2402 38764770 PMC11099385

[10] Bojovic K Simonovic-Babic J Mijailovic Z Milosevic I Jovanovic M Ruzic M Micro-elimination of HCV as a possible therapeutic strategy: our experience and a review of literature. J Infect Dev Ctries. 2020;14:117–24. 10.3855/jidc.11785 32146444

[11] Lazarus JV Safreed-Harmon K Thursz MR Dillon JF El-Sayed MH Elsharkawy AM The Micro-Elimination Approach to Eliminating Hepatitis C: Strategic and Operational Considerations. Semin Liver Dis. 2018;38:181–92. 10.1055/s-0038-1666841 29986353

[12] Huang JF Hsieh MY Wei YJ Hung JY Huang HT Huang CI Towards a safe hospital: hepatitis C in-hospital micro-elimination program (HCV-HELP study). Hepatol Int. 2022;16:59–67. 10.1007/s12072-021-10275-7 34850326 PMC8631565

[13] Zheng L Zhang X Nian Y Zhou W Li D Wu Y Multi-disciplinary cooperation for the micro-elimination of hepatitis C in China: a hospital-based experience. BMC Gastroenterol. 2023;23:386. 10.1186/s12876-023-03016-7 37951862 PMC10638763

[14] Hei FX Ye SD Ding GW Pang L Wang XC Liu ZF Epidemiological Analysis on Reported Hepatitis C Cases in China from 2012 to 2016. Biomed Environ Sci. 2018;31:773–6. 30423279 10.3967/bes2018.103

[15] Cui Y Jia J Update on epidemiology of hepatitis B and C in China. J Gastroenterol Hepatol. 2013;28 Suppl 1:7–10. 10.1111/jgh.12220 23855289

[16] He N Hao S Feng G Gao J Kong FJ Ren ZX [Analysis of the factors influencing the elimination strategies with the current status of diagnosis and treatment of hepatitis C in hospital]. Zhonghua Gan Zang Bing Za Zhi. 2021;29:1053–8. Chinese. 34933422 10.3760/cma.j.cn501113-20210119-00034 PMC12814788

[17] Chen B Xu B Cui HY Ma ZH Guo WH Pei LJ Comparison of effectiveness and cost of different HCV testing strategies in high-risk populations in China. J Med Virol. 2024;96:e29433. 10.1002/jmv.29433 38293900

[18] Manns MP Buti M Gane E Pawlotsky JM Razavi H Terrault N Hepatitis C virus infection. Nat Rev Dis Primers. 2017;3:17006. 10.1038/nrdp.2017.6 28252637

[19] Pradat P Virlogeux V Trépo E Epidemiology and Elimination of HCV-Related Liver Disease. Viruses. 2018;10:545. 10.3390/v10100545 30301201 PMC6213504

[20] Chevaliez S Pawlotsky JM New virological tools for screening, diagnosis and monitoring of hepatitis B and C in resource-limited settings. J Hepatol. 2018;69:916–26. 10.1016/j.jhep.2018.05.017 29800630

[21] Salama II Raslan HM Abdel-Latif GA Salama SI Sami SM Shaaban FA Impact of direct-acting antiviral regimens on hepatic and extrahepatic manifestations of hepatitis C virus infection. World J Hepatol. 2022;14:1053–73. 10.4254/wjh.v14.i6.1053 35978668 PMC9258264

[22] Li LM Zhan SY Chi H Deng Y Wang L Wang B [Suggestions on reforming and improving the prevention and treatment system for major epidemic diseases in China]. Zhonghua Liu Xing Bing Xue Za Zhi. 2020;41:981–5. Chinese. 32340092 10.3760/cma.j.cn112338-20200407-00521

[23] Joukar F Mansour-Ghanaei F Soati F Meskinkhoda P Knowledge levels and attitudes of health care professionals toward patients with hepatitis C infection. World J Gastroenterol. 2012;18:2238–44. 10.3748/wjg.v18.i18.2238 22611318 PMC3351775

[24] Korkmaz P Uyar C Ozmen A Toka O Knowledge and attitude of health care workers toward patients with hepatitis C infection. Southeast Asian J Trop Med Public Health. 2016;47:935–44. 29620797

[25] Cekin AH Cekin Y Ozdemir A The level of knowledge of, attitude toward and emphasis given to HBV and HCV infections among healthcare professionals: data from a tertiary hospital in Turkey. Int J Occup Med Environ Health. 2013;26:122–31. 10.2478/s13382-013-0077-3 23532821

[26] Alzahrani MS Ayn Aldeen A Almalki RS Algethami MB Altowairqi NF Alzahrani A Knowledge of and Testing Rate for Hepatitis C Infection among the General Public of Saudi Arabia: A Cross-Sectional Study. Int J Environ Res Public Health. 2023;20:2080. 10.3390/ijerph20032080 36767451 PMC9915280

[27] Adinolfi LE Nevola R Rinaldi L Romano C Giordano M Chronic Hepatitis C Virus Infection and Depression. Clin Liver Dis. 2017;21:517–34. 10.1016/j.cld.2017.03.007 28689590

