# Supplementary material for: Innovative management of hepatitis C: implementation and outcomes of the ‘SMART’ model in a general hospital
Source: J Glob Health. 2026 Aug 28;16:04241. doi: 10.7189/jogh.16.04241 (PMC13522902; doi:10.7189/jogh.16.04241)
Supplement: Supplementary file 1 [file jogh-16-04241-s001.pdf]

Supplement to: Li M, Hu W, Dai Z, Li J, Ma J, Li Y. Innovative management of hepatitis C: implementation and outcomes of the 'SMART' model in a general hospital. J Glob Health. 2026;16:04241.

Table S1. Baseline Characteristics of HCV Antibody-Positive Patients at The Second Xiangya Hospital of Central South University from January 2020 to December 2023

	**HCV RNA Testing Rate**	χ^2	***P*-value**	**DAAs Treatment Rate**	χ^2	***P*-value**
**Age (years)**						
＞60	60.73% (815/1342)	7.33	0.062	58.55% (161/275)	15.69	＜0.001
40-60	64.50% (1545/2395)			69.88% (413/591)		
＜40	65.70% (406/618)			75.76% (100/132)		
**Gender**						
Female	62.37% (1230/1972)	2.02	0.155	70.33% (256/364)	2.04	0.153
Male	64.46% (1536/2383)			65.93% (418/634)		
**Department visited**						
Infectious diseases	87.91% (865/984)	438.06	＜0.001	84.54% (328/388)	89.63	＜0.001
Others	56.39% (1901/3371)			56.72% (346/610)		
**Patient type**						
Inpatient	62.70% (1798/2866)	2.39	0.122	61.58% (384/623)	26.74	＜0.001
Outpatient	65.08% (968/1489)			77.39% (290/375)		

Table S2. Comparison of HCV Testing and Treatment Rates by Clinical Department Before and After Implementation

	No Management	Micro-elimination	SMART	χ^2	P-value
**RNA Testing rate**					
Infectious Diseases Department	86.58% (258/298)	88.83% (318/358)	88.11% (289/328)	0.81	0.668
Others	44.47% (470/1057)	50.69% (623/1229)	74.47% (808/1085)	222.56	< 0.001
**DAAs Treatment rate**					
Infectious Diseases Department	83.19% (94/113)	79.08% (121/153)	92.62% (113/122)	9.75	< 0.01
Others	52.80% (85/161)	51.70% (91/176)	62.27% (170/273)	6.24	< 0.05

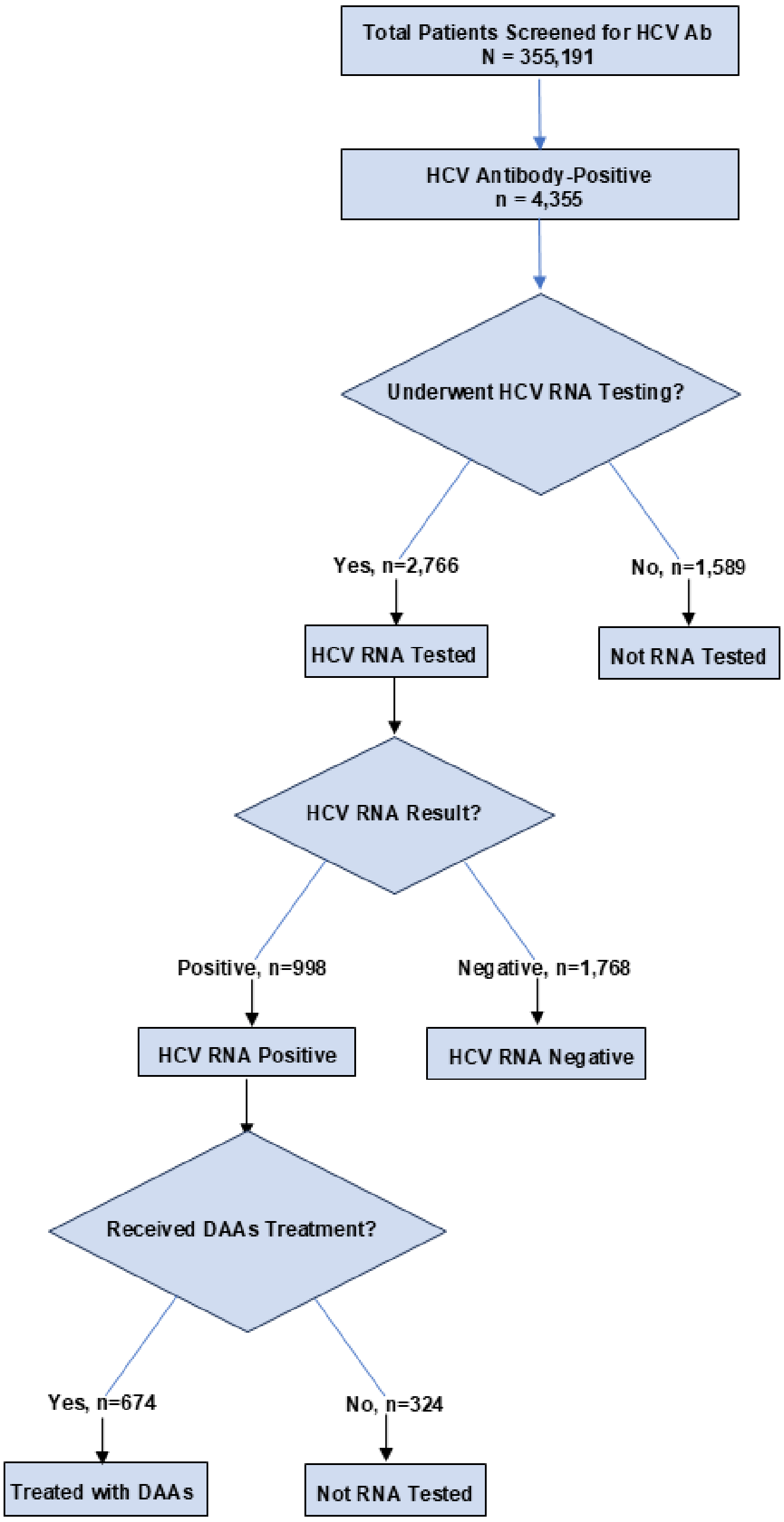

Figure S1. Flowchart

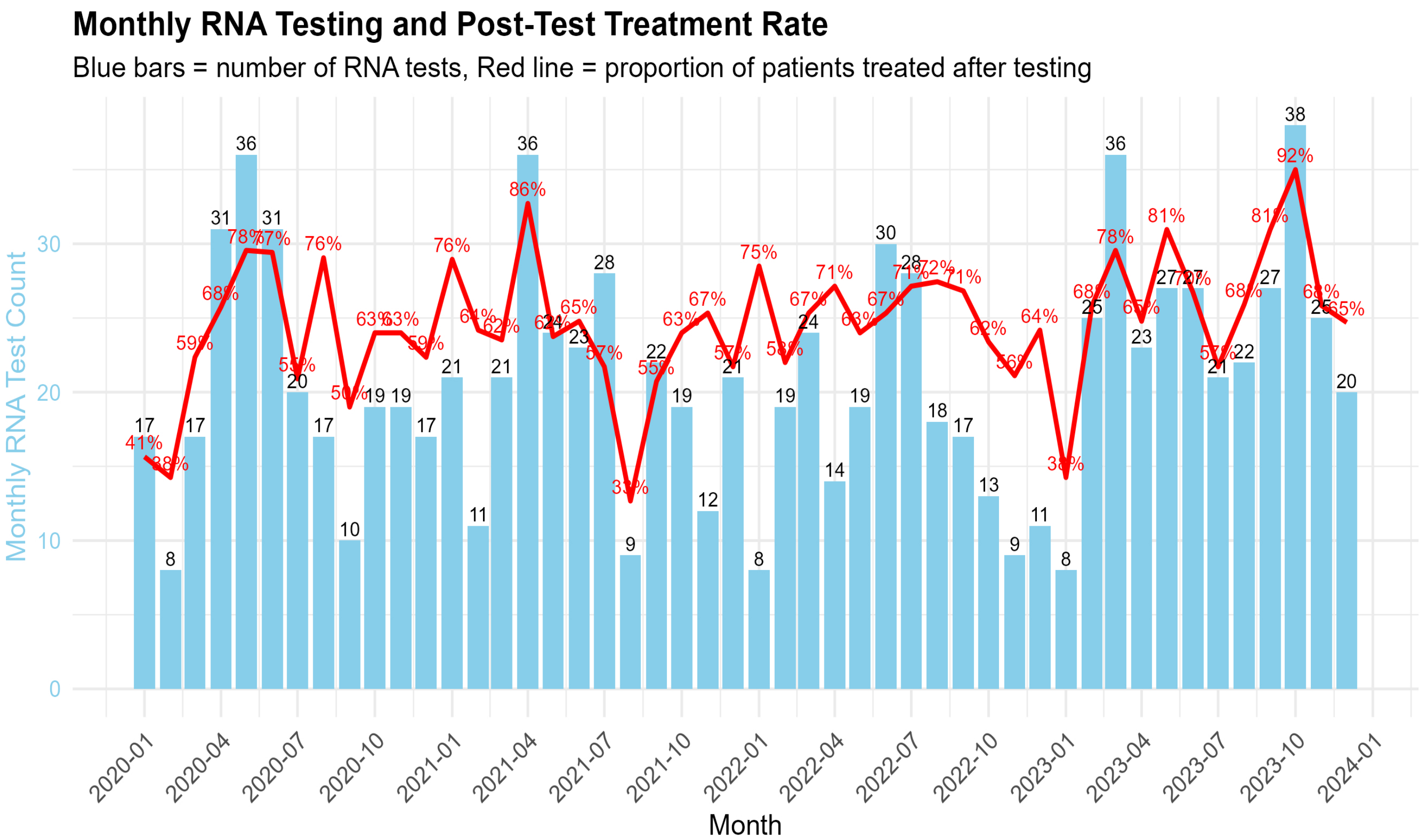

Figure S2. Monthly HCV RNA testing and post-test DAA treatment rates (sensitivity analysis), 2020 – 2023. Blue bars represent the number of patients undergoing HCV RNA testing each month, and the red line represents the proportion of patients who received DAA therapy after RNA testing. Vertical dashed lines indicate the implementation of the micro-elimination strategy (March 2021) and the SMART management model (July 2022). This figure illustrates the monthly trends used to assess the robustness of the SMART model's impact on RNA testing and treatment rates.
